# The Disappearance of Black Men From Medicine: A Consequence of Racism and the Flexner Report

**DOI:** 10.31486/toj.24.5050

**Published:** 2024

**Authors:** Tamika Webb-Detiege

**Affiliations:** Department of Rheumatology, Ochsner Clinic Foundation, New Orleans, LA and The University of Queensland Medical School, Ochsner Clinical School, New Orleans, LA

In 1940, Black men represented 2.7% of physicians in America, and almost 80 years later in 2018, the percentage remained essentially unchanged at 2.6%.^1^ The percentage of Black male physicians continues to remain below the percentage of Black Americans, and many ask, “Why are Black men not becoming doctors?” The approach to answering this question has focused on what Black men lack and need to be given in order to increase their numbers in medical school. However, a deeper dive into our history and the history of medical education is needed to understand the reasons why there are low numbers of Black physicians as a whole and why there has not been a significant increase in the number of Black males entering medical school.

The preamble to the Declaration of Independence states, “We hold these truths to be self-evident, that all men are created equal, that they are endowed by their Creator with certain unalienable Rights, that among these are Life, Liberty and the pursuit of Happiness.” I memorized this sentence in elementary school, but it was not what came to mind when I had my son 16 years ago.

A beautiful healthy baby boy was born to me, a woman classified in America based on the social construct of race as Black. Therefore, my son was classified as Black. “Black male” is typed on his birth certificate. Society will put restrictions on him based on this category. Don’t take this the wrong way; I am proud of my ancestry and of all those who paved the way for me. I also know that there is more that needs to be done.

My wonderful son is the offspring of two physicians. He is not only handsome but smart. He loves reading, science, and math and was named a member of the National Honor Society at his high school. We have been able to keep him safe; however, it has been challenging. Society in America, from his birth to about age 9, saw him as a cute little boy who was intellectually gifted and inquisitive, but something changed around age 10. He went from being seen as a darling sweetheart of a boy to a potential threat. This is a transition that many Black mothers and fathers fear for their sons as well as their daughters. The weight of this perception and other forms of racism is so heavy that it causes premature biologic aging beyond a person's chronologic age in a process known as *weathering*.^2^

Black children begin to experience weathering early in life. Society starts to adultify them as early as preschool, and instead of treating them as children to teach, academic expectations are lowered and they are seen as potential future threats that need to be managed.^3,4^ This adultification leads to excessive discipline in schools and tougher consequences than white juveniles experience when they make a “silly” childhood mistake that leads them to be introduced to the judicial system.^5^

With the adultification, excessive discipline, and lower expectations of Black children, it seems obvious why there would be differences in Black females and males moving forward to become doctors compared to non-Black students, but there is more to this issue than many may want to consider. It includes the very foundation of our current medical education system. In 1910, Abraham Flexner released the Flexner Report that was commissioned by the Carnegie Foundation under the initiation of the American Medical Association, an organization whose local chapters prohibited Black doctors from being members well into the late 1960s.^6^

The Flexner Report is a book-length report on medical education in the United States and Canada that contributed to the evolution of medical education but also helped dismantle medical education opportunities for Black Americans and ultimately assisted in the progression of health disparities we see today.^7^ In his report, Flexner reinforced the notion of *segregated and unequal* for Black medical students. He recommended the closure of 5 of the 7 medical schools for Blacks in the United States. He also encouraged that Black doctors not be trained as surgeons but instead be educated in hygiene to manage the spread of infections that Black people suffer and prevent them from contaminating their “white neighbors.”^8^ Flexner's 2-page chapter “The Medical Education of the Negro” was the nail in the coffin for the future of many Black doctors and the hopes for equitable health for the Black community.

The loss of future Black physicians as a result of Flexner's recommendations that led to the closure of 5 historically Black medical schools has been studied. Campbell et al reported that these schools might have trained between 27,773 and 35,315 physicians between their years of closure and 2019. In 2019 alone, there would have been an additional 355 Black graduating physicians, a 29% increase in the actual number.^9^

Tens of thousands of future Black physicians disappeared as a result of the Flexner Report.^10^ This loss of potential Black physicians is of specific importance to Louisiana. Flint Medical College of New Orleans University, open from 1889 to 1911, was one of those historically Black medical schools that closed following the Flexner Report. This closure still directly impacts the health of the residents of the city of New Orleans, the state of Louisiana, and the Gulf South region who would have benefitted from the medical care these potential doctors would have provided. While we have Abraham Flexner to thank for improving the structure and quality of medical education, we must also acknowledge how his report with its segregationist and biased views has contributed to the lack of Black doctors—both male and female—and the health disparities we see in society, especially in the Gulf South.

Given these 2 major influences on the disappearance of Black male physicians—racism and the closure of historically Black medical schools—where do we go from here? Begin by examining your own biases against Black boys and men; speaking up when bias is expressed by colleagues, friends, and family; and voting in ways that do not support or advance biased agendas in politics. However, intellectual activism is not enough. Systemic changes are also needed. The education system can train teachers to keep their biases from impacting the Black students they teach with regard to discipline and lower expectations. Law enforcement and judicial employees should receive bias training to prevent overpolicing and harsher sentencing of Black children. Current medical schools should accept and support minority students, create a culturally aware environment that values different perspectives and backgrounds, and train physicians to serve diverse communities in an equitable fashion to potentially, through doctor-patient interactions, inspire young Black males to enter the field of medicine. The private sector can make a difference by financially supporting the development of additional historically Black medical schools and providing funds for pipeline programs that work to increase the numbers of Black physicians.

Change is possible. My grandmother's grandfather was enslaved in America. It is also true that my grandmother's granddaughter is now a physician in America. To move the needle on the forces that prevent progress in increasing the number of Black males in medicine, we must all do our part. Being a physician is a wonderful career, and all children deserve the opportunity to realize that goal if they choose.
